# Aberrant splicing in human cancer: An RNA structural code point of view

**DOI:** 10.3389/fphar.2023.1137154

**Published:** 2023-02-23

**Authors:** Maria Apostolidi, Vassiliki Stamatopoulou

**Affiliations:** ^1^ Agilent Laboratories, Agilent Technologies, Santa Clara, CA, United States; ^2^ Department of Biochemistry, School of Medicine, University of Patras, Patras, Greece

**Keywords:** aberrant splicing, pre-mRNA processing, RNA motifs, cancer, RNA G-quadruplexes, RNA structural code, transcriptome diversity

## Abstract

Alternative splicing represents an essential process that occurs widely in eukaryotes. In humans, most genes undergo alternative splicing to ensure transcriptome and proteome diversity reflecting their functional complexity. Over the last decade, aberrantly spliced transcripts due to mutations in *cis*- or *trans*-acting splicing regulators have been tightly associated with cancer development, largely drawing scientific attention. Although a plethora of single proteins, ribonucleoproteins, complexed RNAs, and short RNA sequences have emerged as nodal contributors to the splicing cascade, the role of RNA secondary structures in warranting splicing fidelity has been underestimated. Recent studies have leveraged the establishment of novel high-throughput methodologies and bioinformatic tools to shed light on an additional layer of splicing regulation in the context of RNA structural elements. This short review focuses on the most recent available data on splicing mechanism regulation on the basis of RNA secondary structure, emphasizing the importance of the complex RNA G-quadruplex structures (rG4s), and other specific RNA motifs identified as splicing silencers or enhancers. Moreover, it intends to provide knowledge on newly established techniques that allow the identification of RNA structural elements and highlight the potential to develop new RNA-oriented therapeutic strategies against cancer.

## 1 Introduction

Alternative splicing holds a central role in cellular metabolism and is considered a hub for cell differentiation and tissue development through the diversification of both the proteome and transcriptome (Kaliatsi et al., 2020; Khan et al., 2021; Reixachs-Solé and Eyras, 2022). It occurs in most human genes, with an estimated percentage exceeding 90% (Xiao and Lee, 2010). Along with multiple ribonucleoprotein complexes and auxiliary protein factors that are essential for the integrity of splicing, *cis*-splicing elements are required to drive efficient splicing. Therefore, U1 snRNP complex recognizes an AGGURAGU sequence which corresponds to the 5′ splice site (splicing occurs after AG), while U2AF binds to the 3′ splice site that contains a polypyrimidine tract rich in uracils followed by an AG dinucleotide after which 3′ splicing takes place. An additional branchpoint sequence is located 18–40 nts upstream of the 3′ splice site where an adenine serves as the essential nucleophile that starts splicing after U2 snRNP anchoring (Wahl et al., 2009). Due to accumulating data from whole exome sequencing, numerous *cis*-splicing elements have been identified throughout exons that can promote or inhibit the splicing of specific genes *via* interactions with serine/arginine-rich (SR) proteins or heterogeneous nuclear ribonucleoproteins (hnRNPs), respectively (Busch and Hertel, 2012; Jeong, 2017).

Various mutations either in *cis*-splicing elements, central or accessory proteins of the splicing machinery have been recently linked to deviations in alternative splicing mechanisms and are strictly associated with human diseases, including cancer (Lee and Abdel-Wahab, 2016; Sebestyén et al., 2016; Murphy et al., 2022). A growing number of cancer-related genetic mutations located in *cis*-acting splicing elements of exons or introns, such as E/ISEs (exon/intron splicing enhancers) and E/ISSs (exon/intron splicing silencers), appear to be responsible for inverting their silencer or enhancer actions or generating new splicing element functions (Shiraishi et al., 2018). Such events often result in aberrant splicing and differential recognition by splicing factors, rendering splice variants that dominate tumor-related signaling and metabolism (Coltri et al., 2019). For instance, mutations in splicing elements in exon 18 of the BRCA1 tumor suppressor mRNA change the recruitment and binding mode of specified splicing factors and exclude this exon. Exon 18 contains one of the core BRCA1 C-terminal (BRCT) domains that facilitate binding to target proteins, and therefore, the produced truncated protein is not functional (Goina et al., 2008). In parallel, whole-genome sequencing and RNA-seq data allowed the identification of multiple intronic mutations that can alter the splicing fidelity and cause human cancer initiation, with blood cancers ranking at the top of the list (Jung et al., 2021). Most interestingly, except for intronic mutations close to the splicing sites, it has been reported that mutations even 10 kb far from exon-intron junctions can also create new splicing sites leading to partial intron retention or pseudoexon activation in cancer (Jung et al., 2021).

Apart from short single-stranded RNA sequences acting as *cis*-regulatory elements and various RNA binding factors influencing the splicing cascade, RNA structures are now emerging as essential splicing regulators (Chen and Manley, 2009). Although several RNA motifs have been identified as exonic silencers or enhancers, our understanding of RNA structures as splicing regulatory elements is still inadequate. Recent studies sought to determine the code that dictates splicing mechanisms and coordinates alternative splicing with a basis of not only the RNA sequence but also the RNA structure (Rosenberg et al., 2015), implying an additional layer of regulation. In addition, accumulating evidence supports that exonic inclusion could be determined not only by the splicing cascade but also by the transcriptional rate, based on data that propose coupling of transcription with splicing (Close et al., 2012; Naftelberg et al., 2015). Hence, the existence of an RNA structural code may suggest a common underlying regulatory mechanism that governs both processes.

In this short review, we describe an overview of the RNA structural elements that are recently proven to shape the alternatively spliced transcriptome and their implication in tumorigenesis, focusing on G-quadruplex motifs. Moreover, we provide available knowledge on bioinformatic tools and current experimental methodologies that could be exploited separately or in combination to efficiently detect such RNA structures and deeply understand their involvement in alternative splicing. Finally, we include an RNA-oriented therapeutics section hinting at anti-sense oligonucleotide (ASO)- and small molecule-based strategies that could potentially repair specific pre-mRNA aberrant splicing and treat the underlying pathologies with a focus on aggressive cancer.

## 2 Alternative splicing modulation by RNA G-quadruplexes

RNA G-quadruplexes (rG4s) are secondary RNA structures formed by guanine-rich sequences organized in stacks of G-quartets promoted by Hoogsteen hydrogen bonds between guanines (Fay et al., 2017). G4 structures are arraying throughout the pre-mRNA transcript body and are most frequently located in high densities in both 5′ and 3′ untranslated regions (UTRs) and close to splicing sites (Huppert et al., 2008; Chambers et al., 2015). Selected cations, such as K^+^, further stabilize the formation of rG4s by fitting the space between two G-quartets (Kwok and Merrick, 2017; Kwok et al., 2018). rG4s are emerging in the limelight of gene expression regulation as essential elements that form functional structures in human cells. Their intracellular folding appears to impact several post-transcriptional processes, including pre-mRNA processing (such as alternative splicing), mRNA turnover and translation (Marcel et al., 2011; Beaudoin and Perreault, 2013; Yu et al., 2014).

In addition to various bioinformatic approaches that have been developed to predict and verify the existence of G4 structures in the genome (Huppert and Balasubramanian, 2005; Todd et al., 2005; Bedrat et al., 2016), several experimental data demonstrate the formation and function of rG4s *in cellulo* (Biffi et al., 2014; Cammas et al., 2015; Thandapani et al., 2015; Xu et al., 2015). Notably, recent research suggests the implication of those elements in underlying regulatory mechanisms that control the expression of hallmark genes associated with cancer (Cammas and Millevoi, 2017). Moreover, *in vivo* mapping of rG4s contemplates the recruitment of specific RNA binding proteins (RBPs) that recognize, bind, and unwind RNA G4 structures (Guo and Bartel, 2016). Along the same lines, concomitant RNA-RNA interactions could facilitate sequestration mechanisms attributed to the steric hindrance effect around the G4 structure, or tandem rG4s, also described as the molecular crowding effect (Reddy et al., 2013).

RNA G4 structures are more stable than their DNA counterparts (dG4s), suggesting that rG4s plausibly control genome decoding by influencing the transcriptome (Arora et al., 2008; Joachimi et al., 2009). In addition, transcriptome-wide profiling of rG4 structures has demonstrated their affluent dispersion in the human transcriptome (Kwok et al., 2016). In contrast to dG4s, the modulatory effect of rG4s is more prevalent and attributed to the single-stranded nature of RNA that favors the formation of secondary structures. Compared to DNA which is confined to the nucleus, rG4s are present in both the nucleus and the cytoplasm and, therefore, they are accessible for binding to a diverse pool of regulatory factors (Fay et al., 2017). Additional evidence supports that except for their favorable formation in cells, rG4 forming sequences could accommodate a variety of secondary structural formations beside the canonical, indicating a complex layer of regulation based on differential cellular states (Pandey et al., 2015; Chow et al., 2020).

Although there are hints in the literature suggesting the involvement of rG4s and other RNA structures in alternative splicing, a unanimous functional mechanism remains controversial (Warf and Berglund, 2010; Weldon et al., 2018; Zhang et al., 2019). In more than a quarter of human genes, rG4 motifs are abundant in splicing junctions or at close distance to them. rG4s in these positions are associated with alternative splicing plasticity in response to dramatic changes in cell physiology, such as differentiation or phenotypic change. Exon skipping due to rG4 presence at the exon’s splice sites is a frequent paradigm that elucidates rG4 impact on alternative splicing (Georgakopoulos-Soares et al., 2022). Moreover, rG4s located in the vicinity of purine-rich single-stranded splicing enhancers are sequestering their recognition by SR splicing regulatory proteins and impede their binding, which, in turn, alternate the processing of adjacent splice sites (Millevoi et al., 2012). On the other hand, two distinct G4 structures in exon 15 within the fragile X mental retardation protein 1 (FMR1) mRNA act as splicing enhancers where FMRP can bind and promote the exon splicing (Didiot et al., 2008).

While rG4s within intronic regions appear to modulate splicing by promoting exons inclusion (Huang et al., 2017), a G4 element located in intron 6 of the human telomerase reverse transcriptase (hTERT) transcript acts as a splicing silencer facilitating exon skipping and the domination of spliced variants encoding for inactive hTERT protein (Gomez et al., 2004). Μoreover, rG4s positioned in weak splice sites can dictate exon recognition (Georgakopoulos-Soares et al., 2022). Overall, mutations in G4 motifs and disturbance of their structure could lead to dramatic changes in the hierarchic inclusion or exclusion of exons or introns with detrimental clinical outcomes.

Proteome-wide pull-down experiments coupled with mass spectrometry have identified several hnRNPs and splicing factors that bind G4 structures in pre-mRNAs and alter the splicing regulation presets (von Hacht et al., 2014). Additional integrated analysis of enhanced crosslinking and immunoprecipitation assays (eCLIP) and loss of function (LoF) RNA-seq experiments have verified a list of proteins that modulate alternative splicing by binding to G4 motifs. For instance, the hnRNPK/U ribonucleoproteins exhibit high affinity to G4 motifs proximal to splice sites and positively regulate exon inclusion (Georgakopoulos-Soares et al., 2022). FMRP protein, a shuttle required for mRNA transportation, binds to G4 elements of its own transcript and enhances its splicing (Didiot et al., 2008). Moreover, most AFF transcription activator family members can bind to rG4 structures and impact alternative splicing by modulating exon inclusion. Non-canonical expression of AFF proteins and impeding alterations in the splicing of their transcript targets are associated with X-linked intellectual disability (Melko et al., 2011). On the other hand, rG4s could also prevent specific splicing factors, such as the AQR spliceosomal factor, from binding around splice sites leading to the inhibition of alternative exon inclusion (Georgakopoulos-Soares et al., 2022).

## 3 Cancer-related aberrant splicing driven by RNA motifs

The importance of RNA structural elements in shaping the alternatively spliced transcriptome in tumorigenesis was highlighted recently (Zhang et al., 2021). Moreover, the prevalence of G4 structures within the human transcriptome hints at their significant involvement in splicing regulation suggesting a major modulatory role of rG4s in cancer. Single nucleotide polymorphisms (SNPs) within G4 sequences can disrupt their secondary structure leading to altered splicing events often in cancer-related genes and in turn to tumorigenesis or tumor progression and metastasis (Beaudoin and Perreault, 2010). For instance, an rG4 located in intron 3 of the TP53 transcript interacts with splicing factors and promotes the splicing of intron 2. However, an SNP polymorphism within the G4 sequence modifies the binding capacity of ligands resulting in the retention of intron 2 and the expression of elongated TP53 variants that dominate over the fully spliced isoform. Increased expression of such variants leads to impairment of TP53 functions in suppression of cell growth and thus controlling tumor formation and progression (Perriaud et al., 2014).

**FIGURE 1:**
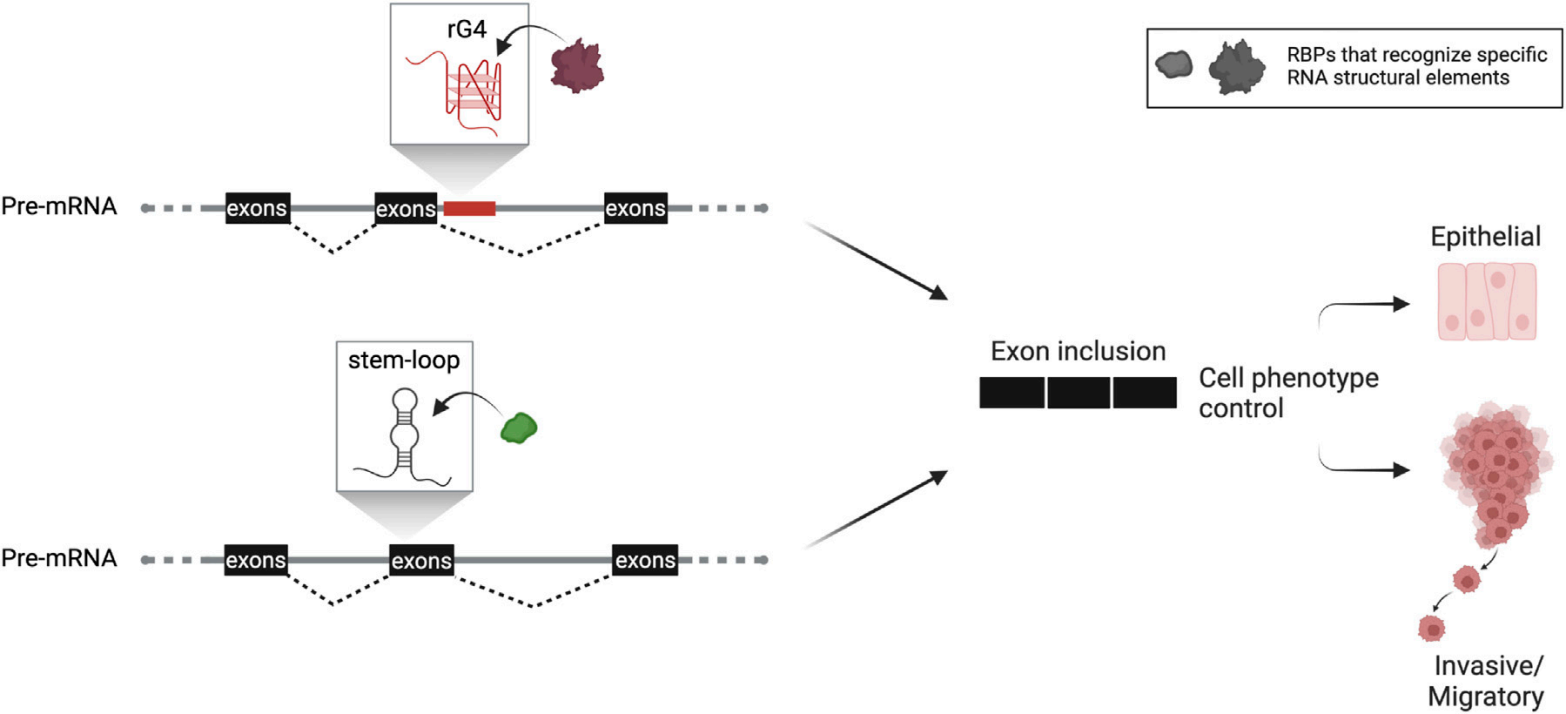
Hypothetical scheme of RNA structure-based control of alternative splicing adapted from work described by Huang et al. (2017) and Fish et al. (2021). Based on this knowledge, RNA structures (either G4 or simpler stem-loop structures) confined in the vicinity of splice sites are implicated in alternative splicing dictating cancer cell phenotypic plasticity. This critical process promotes tumorigenesis and metastasis and is tightly linked with the patient’s prognosis. Both structural elements, rG4 or stem-loop structure, are used as splicing enhancers that include the regulated exon or exon cassette. The rG4 is close to the splice site in the downstream intron or within the regulated exon cassette, as described for stem-loop structure. Both are recognized by specific spliceosomal complex’s factors or auxiliary proteins. Formation of such RNP complexes then leads to an alternate spliced protein variant that marks the cancer cell’s phenotypic fate.

Epithelial-to-mesenchymal transition (EMT) is an essential process in cancer that cultivates stem-like properties in cancer cells, dictating phenotypic plasticity during tumorigenesis and metastasis. It has been reported that an rG4 in intron 8 of CD44, a marker for cancer cell stemness, regulates the inclusion of the upstream variable exon 8. Expression of this CD44 variant maintains the epithelial phenotype (Huang et al., 2017), indicating a direct connection between G4-mediated alternative splicing regulatory mechanism and phenotypic plasticity during EMT in cancer (Figure 1). Similar cases have been described in the literature, demonstrating the implication of intronic rG4s in splicing efficiency (Huang et al., 2017; Verma and Das, 2018). Nonetheless, further transcriptome-wide investigation on additional G4-mediated mechanisms will display the extent of rG4s′ impact on alternative splicing and their implication in cancer.

Besides G4, an additional RNA structure has been described in enhancing splicing in highly metastatic cancer cells. Specifically, Fish et al. (2021) identified by combining RNA-seq and *in silico* folding software an SSE (structural splicing enhancer) in the pre-mRNAs of PLEC which encodes plectin, a cytoskeletal protein essential for tissue integrity, and ERRFI1, a negative regulator of EGFR signaling. This SSE is located near cassette exons, and due to its high composition of GCs, it folds into a stable stem bearing a small bulge around the fifth nt on the top of the stem. The stability of the SSE secondary structure is crucial to interact directly and in a primary sequence-independent manner with SNRPA1, a component of U2 snRNP that is responsible for the branchpoint recognition site in the initial steps of pre-mRNA splicing. Intriguingly, it has been reported that SNRPA1, beyond its nodal role as the core of U2 complex, acts as a regulator of PLEC and ERRFI1 alternative splicing by interacting with SSEs. Notably, this specific interaction promotes the invasion and migration of breast cancer cells resulting in metastatic lung colonization, while SNRPA1 upregulation is associated with a poor prognosis of the disease (Fish et al., 2021) (Figure 1).

### 3.1 The PKM case: A unique event of mutually exclusive exon-switching essential for cancer metabolism

PKM2 is the splicing variant of pyruvate kinase that correlates with cancer. In most tumor cells, the switch from PKM1 to PKM2 isoform offers new metabolic wiring towards aerobic glycolysis, resulting in increased glucose consumption that characterizes highly proliferative cells (Vander Heiden et al., 2009; Zahra et al., 2020). Alternate inclusion of exon 9 or 10 promotes the expression of either isoform (Christofk et al., 2008). cMyc oncogenic factor regulates the expression of certain hnRNPs (hnRNPA1, hnRNPA2, and PTB), leading to induction of PKM pre-mRNA alternative splicing and concomitant expression of the PKM2 isoform that supports cancer metabolism (Clower et al., 2010; David et al., 2010). hnRNPA1 orchestrates the binding of hnRNPA2 and PTB suppressor proteins in RNA elements flanking in or surrounding exon 9, resulting in PKM2 transcript production by synergistically excluding exon 9 (David et al., 2010). Lowering the expression level of these factors alters RNA motif binding selectivity and shifts to exon 9 inclusion and exon 10 exclusion in most instances, fostering the expression of PKM1 isoform (Chen et al., 2012).

## 4 Detection and analysis of RNA secondary structures

Within the last decade, the increased need for mapping G4 or analogous structures within the human transcriptome in order to understand their functional relevance in RNA processing and turnover has led to the development of various detection tools and analysis methods. Apart from genome- and transcriptome-wide studies focusing on the reconstitution of G4 structure *in vitro*, probing such structures *in vivo* still draws attention. Most available analogues or fluorescent probes target DNA G4 structures (dG4s), such as pyridostatin analogues that stabilize telomeric dG4s or the recently developed G4-specific fluorescent probe (SiR-PyPDS) used for live imaging analysis of individual G4 structures in cells. Additional evidence suggests that universal G4 probes, such as G4-specific antibodies BG4, can detect fully folded and stable dG4 and rG4 structures equally. In addition, a recent study showcases a next-generation quadruplex ligand (N-TASQ) to probe rG4 structures in untreated living cells (Müller et al., 2012; Laguerre et al., 2015; Di Antonio et al., 2020; Summers et al., 2021).

On the other hand, several algorithms or web-based platforms can detect G4 structures within a given DNA or RNA sequence. Initial versions of such tools, such as the QGRS mapper (Kikin et al., 2006), can predict G4 formation within G-rich sequences based on similarity to G4 sequences and the likelihood of forming stable G4 structures. More recent tools, such as the G4Hunter (Bedrat et al., 2016), have also incorporated the ability to detect the propensity of G4 formation, including the prediction of both putative and non-canonical G4s by matching other additional physicochemical characteristics, like melting temperature and spectrometric absorbance. Although these tools are practical for scanning the transcriptome and identifying plausible locations of RNA structural elements with accuracy, they cannot predict their folding and stability *in cellulo*. Therefore, downstream applications have often been used to verify secondary structural determinants and functionality within predicted motifs. Most of those methodologies used for decades are low throughput, such as circular dichroism spectroscopy, and are employed to recapitulate predicted secondary structures within transcripts by reconstituting their folding *in vitro*.

In recent years the widespread usage of highly sensitive RNA-seq methodologies led to revisiting existing low throughput RNA analyses. That resulted in the development of combinatory approaches that leverage the classical RNA structural detection to be applicable in complex RNA sequence mixtures without requiring *a priory* folding prediction (Lyu et al., 2021). These novel high-throughput assays use specific ligands to target and pull-down an enriched pool of RNA structure-containing transcriptome. For instance, the G4RP-seq methodology targets specifically rG4s and could be used to evaluate the relative abundance of the G4 transcriptome in human cell models (Yang et al., 2018). In parallel, other methodologies that expand previously used footprinting methodologies, such as the recently described Keth-seq, are employed to map redundant or tandem rG4s and additional secondary structures throughout the transcriptome of different cell models (Weng et al., 2020).

## 5 Pharmacological targeting of RNA motifs and the way beyond

As discussed in this review, along with transcriptional, post-transcriptional regulation is at the core of genome decoding. Such events are primarily involved in many diseases, including cancer, calling for the development of novel and efficient RNA-targeted therapies. In this context, ASO-based approaches have come to the limelight to repair aberrantly spliced transcripts and treat various disorders (Potaczek et al., 2016; Quemener et al., 2020). ASOs are short synthetic single-stranded RNA or DNA molecules, about 12–25 nucleotides in length, designed to be complementary to their target sequence (Chan et al., 2006; Bennett and Swayze, 2010). Besides their accurate sequence, ASOs have been designed to bear modifications that facilitate their infusion through the plasma membrane and maximize their resistance against degradation by nucleases and their specificity against the target (Opalinska and Gewirtz, 2002). Based on the type and abundance of modifications, ASOs are classified into three generations. The first generation of ASOs includes modifications in the phosphate backbone that increase their stability and extend their half-life (Potaczek et al., 2016). The second generation bears additional nucleotide-specific modifications in the 2′ position of the ribose, such as 2′-O-methyl (2′-OME) or 2′-O-methoxyethyl (2′-MOE) modified bases, which are used to prevent RNase H mediated cleavage and decrease toxicity (Monia et al., 1993; Nomakuchi et al., 2016). Third-generation ASOs are extensively modified, using different nucleic acid synthesis approaches, such as locked nucleic acid (LNA) analogues that confer high binding affinities to target along with improved pharmacokinetic profiles (Geary et al., 2015; Khvorova and Watts, 2017). ASOs can target alternative spliced transcripts and modify or repair the reading of splicing elements such as splicing enhancers or silencers by masking their sequence to identifiers or prevent splicing proteins from binding to RNA secondary structural elements (Hua et al., 2011; Wheeler et al., 2012). A significant paradigm of an effective ASO-based therapeutic approach was the development of Nusinersen, an FDA- and EMA-approved second-generation ASO. Nusinersen targets an intronic silencer that controls the production of the full-length survival of motor neuron 2 (SMN2) protein rescuing the cells from the loss of its paralog SMN1, a culprit of spinal muscular atrophy (Hua et al., 2011; Mercuri et al., 2018).

As mentioned above, a hallmark of cancer metabolism is an isoform switch of PKM1 to PKM2 caused by a unique, mutually exclusive alternative splicing event (Dayton et al., 2016). Hence, it has been shown that simultaneous downregulation of PKM2 and upregulation of PKM1 could induce apoptosis and suppress tumor progression (Goldberg and Sharp, 2012; Lunt et al., 2015). On top of that, different studies have linked PKM2 and its characterized non-metabolic functions, including its relocation to the nucleus and extracellular secretion, with cancer aggressiveness and promotion of metastasis (Zhu et al., 2017; Wang et al., 2020; Apostolidi et al., 2021). Overall, PKM2 dictates cancer cell’s fate, and it is therefore considered an appealing therapeutic target on which many studies have focused over the years in an attempt to inhibit PKM2 production and function (Anastasiou et al., 2012; Adem et al., 2018). Recent studies have systematically sought *cis*-acting elements within the exclusive exon 10 of PKM2, aiming to force the switch to the PKM1 isoform. One identified exonic splicing enhancer recognized by the SRSF3 splicing activator seems critical for producing the PKM2 splice variant. A second-generation ASO designed to anneal to the enhancer sequence disrupts the interaction with the SRSF3 and diminishes PKM2 production, leading to apoptosis induction in glioblastoma cells (Wang et al., 2012a; Wang et al., 2012b). Further screenings using potent ASO inhibitors that target the exon 10 sequences have indicated additional regions with similar effects (Wang et al., 2012b; Ma et al., 2022). Nevertheless, their intracellular folding status and integration with protein modulators have yet to be verified. A list of third-generation ASOs has been recently designed using locked nucleic acid and DNA mixed chemistries and tested both in cells and *in vivo.* Notably, the increased PKM1 isoform production led to concomitant metabolic rewiring in cancer cells and reduced tumor formation in xenograft mouse models (Ma et al., 2022).

Altogether, ASOs are considered novel and promising therapeutic approaches toward personalized medicine, and various ASO-based treatments are currently in the preclinical stage, including aggressive cancer (Quemener et al., 2020). However, ASO- and CRISPR/Cas-based genome editing clinical approaches used for RNA targeting remain challenging due to complications in delivery and severe side effects requiring further optimization (Luther et al., 2018; Rinaldi and Wood, 2018). In addition, targeting RNA structures is a rapidly evolving field and includes several strategies that combine the use of computational tools with high-throughput assays for identifying, designing, and validating small molecules against specific RNA structural elements found either in bacterial pathogens or human (Childs-Disney et al., 2022). Furthermore, the existence of personalized RNA structural patterns in each individual remains an open question which clarification could open new avenues for establishing precision medicine approaches.

The abundancy of RNA structures within the transcriptome indicates the existence of a structural code that defines an additional layer of accuracy and precision in the modulation of gene expression. Moreover, distinguishable characteristics of this code, including the plasticity of RNA structures and their versatile recognition by RNA-binding proteins, allow for distinctive regulatory responses both at the transcriptional and translational levels. As highlighted in this review, increasing evidence implies that such RNA structural code influences regulatory systems to facilitate metastatic signaling integration, which is critical for cancer progression (Figure 1). Consequently, the demonstration of RNA structure as a central player in alternative splicing modulation during those processes clearly depicts its potential value as means to control cancer cell aggressiveness. However, further advances in detection and functional analysis methodologies, as well as a more intensive investigation of the underlying mechanisms of action, are required to further support the exploitation of RNA structures as appealing pharmacological targets.
